# Tight Junction Proteins and Oxidative Stress in Heavy Metals-Induced Nephrotoxicity

**DOI:** 10.1155/2013/730789

**Published:** 2013-04-22

**Authors:** José L. Reyes, Eduardo Molina-Jijón, Rafael Rodríguez-Muñoz, Pablo Bautista-García, Yazmin Debray-García, María del Carmen Namorado

**Affiliations:** Physiology, Biophysics and Neurosciences Department, Center for Research and Advanced Studies, National Polytechnic Institute, Avenida Instituto Politécnico Nacional 2508, Colonia San Pedro Zacatenco, 07360 Mexico, DF, Mexico

## Abstract

Kidney is a target organ for heavy metals. They accumulate in several segments of the nephron and cause profound alterations in morphology and function. Acute intoxication frequently causes acute renal failure. The effects of chronic exposure have not been fully disclosed. In recent years increasing awareness of the consequences of their presence in the kidney has evolved. In this review we focus on the alterations induced by heavy metals on the intercellular junctions of the kidney. We describe that in addition to the proximal tubule, which has been recognized as the main site of accumulation and injury, other segments of the nephron, such as glomeruli, vessels, and distal nephron, show also deleterious effects. We also emphasize the participation of oxidative stress as a relevant component of the renal damage induced by heavy metals and the beneficial effect that some antioxidant drugs, such as vitamin A (all-trans-retinoic acid) and vitamin E (**α**-tocopherol), depict on the morphological and functional alterations induced by heavy metals.

## 1. Introduction

Kidney plays the most important role in elimination of xenobiotics, including drugs and toxic environmental agents. Due to its secretory mechanisms, tubular proximal cells are often exposed to higher concentrations of toxic substances than those occurring in plasma or extracellular fluids. In addition to these circumstances, kidney depicts several mechanisms to absorb and excrete xenobiotics. They use molecular protein components located at the cell membrane, that are involved in the transport through the epithelial cells (transcellular route) and those located between cells (paracellular route). In this review we focus on the alterations induced by heavy metals on the proteins that participate in the paracellular transport (junctional adhesion molecules, claudins, occludin, and zonula occludens). Awareness of the damage to these proteins and the relevant consequences, it has on renal function and morphology, has been recently developed.

Heavy metals are accumulated in the kidney and liver, for this reason it is more accurate to estimate degree and duration of exposition to them, by measuring their concentrations in renal and hepatic tissues than in blood. Other options to measure them are in nails and hair. Kidney is the target of heavy metals and the proximal tubule has been recognized for a long time, as the main site of accumulation and damage; however, in this review we will describe that other segments of the nephron are also damaged after exposition to them, and that this damage leads to severe alterations in renal function, that may be permanent or reversible.

The toxic effect of heavy metals on human body has been recognized. In the past century due to the development of industrialized world, large amounts of these elements were produced and most of them were not biodegradable, remaining in the environment for long periods of time. The problem of disposal of toxic products is more extended in emerging economies. In Latin America despite of strict legal regulations, high levels of these elements are found in soil and sediments, leading to the risk of chronic exposure in the general population. In addition, in those countries with volcanic areas, such as Mexico, heavy metals are present in fossil waters that are extracted for human and animal use [1, 2].

Some heavy metals are necessary for vital functions of the human body, such as iron (Fe), cobalt (Co), copper (Cu), manganese (Mn), molybdenum (Mb), and zinc (Zn). Although it is unknown whether metals such as lead (Pb), cadmium (Cd), and arsenic (As) have a physiological role, they adversely affect several organs, including the liver, lungs, and kidney, being the latest particularly sensitive to their toxic effects even at low levels, due to its ability to reabsorb and accumulate these divalent metals [3]. The extent of renal damage depends on the nature, the dose, route, and duration of exposition displaying diverse degree of severity ranging from mild tubular dysfunction to severe renal failure.

## 2. Tight Junction (TJ) Proteins in Renal Physiology

### 2.1. Renal Transport through Transcellular and Paracellular Pathways

Reabsorption of filtered solutes and water is one of the vital functions performed by the kidney. It occurs in several segments of the nephron, the functional unit of the kidney. Reabsorption occurs through two pathways, transcellular and paracellular. The transcellular route is dependent on specialized molecules, such as transporters, ionic channels, and water channels. The paracellular reabsorption is mediated by the proteins that form the TJ: junctional adhesion molecules (JAMs), occludin, claudins, tricellulin, and scaffolding proteins zonula occludens (ZO-1, ZO2, and ZO-3), for reviews see [4, 5]. These proteins have differential distribution along the nephron and this distribution determines the paracellular permeability of the respective segments. The magnitude of this transport is variable, according to the specific ion. For instance 90% of the absorption of calcium (Ca^2+^) in proximal tubule occurs through the paracellular route, while in the distal tubule 100% of Ca^2+^ reabsorption occurs through the transcellular route. In Henle's loop 50% is transcellular and 50% is paracellular. In contrast, renal glucose reabsorption is transcellular and it is achieved at the proximal tubule through specific transporters, sodium-glucose cotransporters (SGLT 1 and 2) located at the apical brush border and glucose basolateral transporters (GLUT 1 and 2) [6, 7].

From 60 to 70% of the absorption of the glomerular filtered load of ions, organic compounds, and water occurs in proximal tubules, through both, transcellular and paracellular routes [8].

### 2.2. Distribution of TJ Proteins along the Nephron

**Figure 1:**
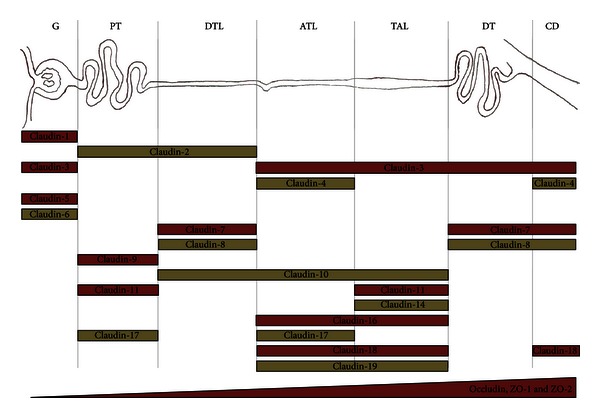
Renal distribution of claudins, occludin, and zonula occludens (ZO-1 and ZO-2) along the nephron. Proteins of tight junctions (TJs) display combined expression patterns along the nephron. G: glomerulus; PT: proximal tubule; DTL: descending thin limb of Henle; ATL: ascending thin limb of Henle; TAL: thick loop of Henle; DT: distal tubule; CD: collecting duct.

Distribution of TJ proteins along the nephron has been shown to be similar in several mammal species, such as mouse, rat, and rabbit, and has been related to the permeability characteristics of the segments that constitute the nephron [9] (Figure 1). This permeability can be estimated by measuring the transepithelial electrical resistance (TER). TER is high in tight epithelia with low paracellular permeability, while in leaky epithelia TER is low. In the nephron this resistance increases from the glomerulus, where no TER has been measured, to the proximal tubule (5 to 6 Ω·cm^2^), and to the collecting tubule that depicts the highest electrical resistance (200 to 600 Ω·cm^2^) [10].

**Figure 2:**
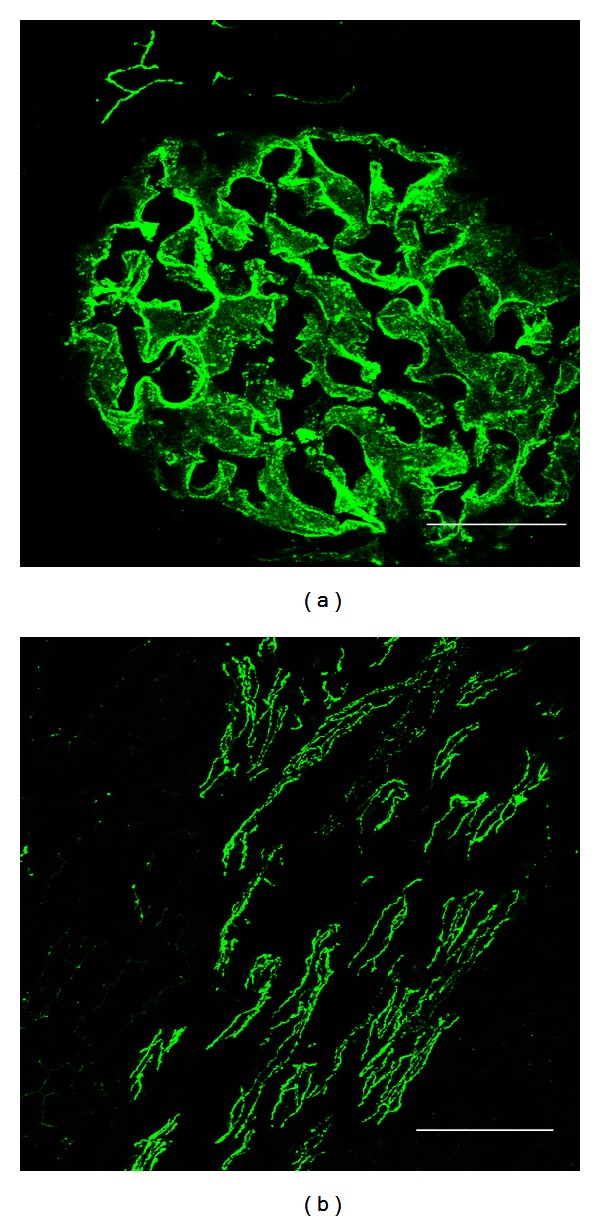
Claudin 5 in glomerulus and in medullary vessels from rat kidney. Claudin 5 is expressed at the endothelial tight junctions (TJs) of glomerular capillaries (a) and in medullary vessels (b), in a renal section from a normal rat. Bar = 50 *μ*m.

**Figure 3:**
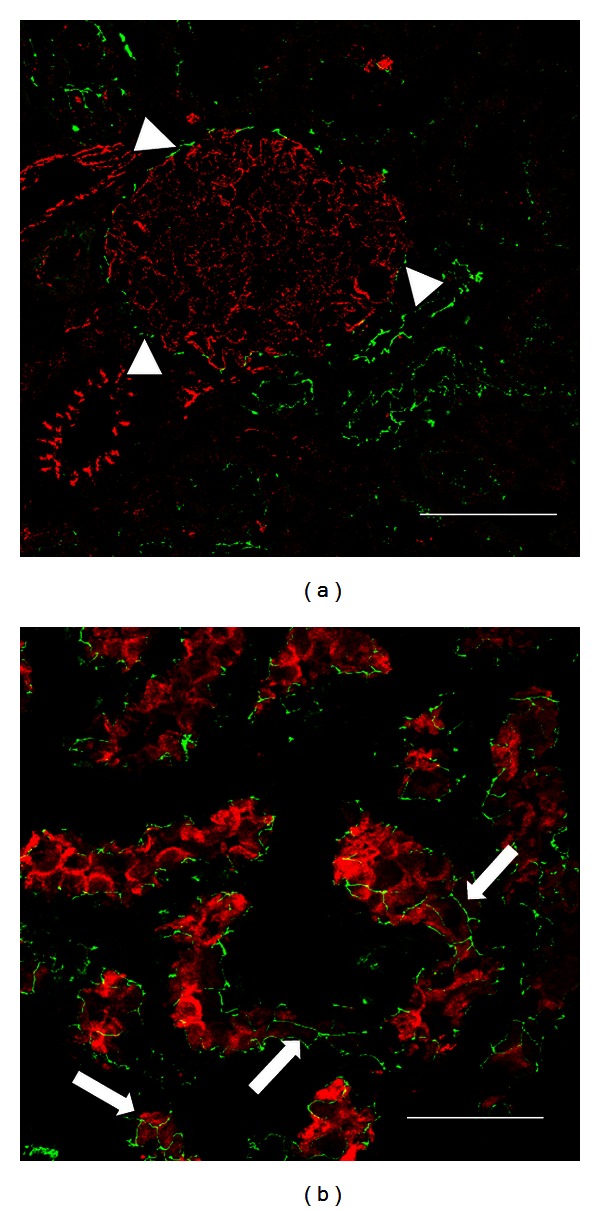
Expression of claudin 2 in Bowman's capsule and proximal tubule in kidney from rat. In renal sections from a normal rat, claudin 2 (green label) costained with claudin 5 (red label), is expressed in Bowman's capsule (arrow heads, (a)). Claudin 2 (green label) costained with dipeptidyl peptidase (red label) shows a typical “chicken fence” pattern in proximal tubules (arrows, (b)). Bar = 50 *μ*m.

*Glomerulus.* Claudin 5, an endothelial TJ protein, has been identified in glomeruli and vessels [11] (Figure 2). Also claudin 3 is located at glomerular capillaries. In Bowman's capsule, an epithelial structure, claudin 2 has been observed (Figure 3(a)). 

*Proximal Tubule.* Claudin 2 is the most abundant of the claudin's family (formed by more than 20 isoforms) in the proximal tubule (Figure 3(b)). This claudin has been recognized as a cation and water channel [12, 13]. It has been located in other highly permeable epithelia that depict low TER [14]. The location of claudin 2 is in agreement with the characteristics of the paracellular pathway in the proximal tubule, which is the most permeable tubular segment of the nephron [15]. Claudins 1, 9, 11, and 17 are expressed in this tubular segment. Occludin, ZO-1, and ZO-2 are also located along the nephron, with increasing amounts from proximal tubule to collecting duct [11] (Figure 1). 

*Thin Descending and Ascending Loops of Henle.* In descending loop of Henle claudins 7 and 8 are located at the aldosterone-sensitive distal nephron and at descending and ascending thin segments [16, 17]. In contrast to proximal tubule, claudin 2 is not present at these segments. 

*Thick Ascending Loop of Henle.* In this segment the role of claudin 16 has been identified as a critical factor for the paracellular absorption of Ca^2+^ and magnesium (Mg^2+^) [18]. Claudins 11 and 14 are also in this segment [19]. 

*Distal Tubule.* In this segment the presence of claudin 3, 4, and 8 has been reported. These three proteins have a role as barriers for cations absorption [15]. 

*Collecting Duct.* This is the segment that depicts the highest electrical resistance in the nephron. Claudins present in collecting tubules share the property of conferring low ionic paracellular permeability, especially to cations. It is noteworthy that expression of claudin 8, present in this segment, has been reported to be augmented by aldosterone. Claudins 4, 7, and 18 are also present in collecting tubules, as well as occludin, ZO-1, and ZO-2 [9] (Figure 1).

In general terms, distribution of claudins along the nephron follows the pattern of TER present in this structure. It increases from the proximal tubule to collecting duct and in opposite direction, paracellular permeability decreases from proximal to collecting duct [11, 20, 21].

## 3. Nephrotoxicity of Heavy Metals on TJs

### 3.1. Absorption and Metabolism of Divalent Metals

The absorption of heavy metals, such as Cd and Pb, is carried out in the small intestine by a divalent metal transporter characterized as DMT-1. This transporter is expressed in the duodenum, red blood cells, liver, and in the proximal convoluted tubular cells of the kidney. This protein transports Fe and displays a high affinity for other divalent metals such as Cd, Nickel (Ni), Pb, Co, Mn, Zn, and Cu [22].

Once absorbed, heavy metals are accumulated in liver where they bind to metallothioneins (MTs). These proteins are widely expressed through the body and have a particular feature; they contain a large quantity of cysteine, which confers both a high affinity and a storage capability to heavy metals, such as Zn, Cd, Mercury (Hg), Cu, Pb, Ni, Co, and Fe. The main role of MT is to transfer heavy metals to metalloproteins, transcription factors, and enzymes [23].

### 3.2. Cadmium

Cadmium is one the most important toxic elements to which general population is exposed. The most common exposition circumstance occurs by contact with tobacco smoke, contaminated water, and foodstuffs such as vegetables, grains, and molluscs. Cadmium progressively accumulates in the body compartments, notably in liver and kidney, given rise to its long half-life, which is more than 20 years in human [24].

Once absorbed and dissociated from MT by the action of gastric environment, Cd binds to albumin and then it is transported to liver, where it binds to glutathione (GSH) and MT-1. This complex has a low molecular weight (<7 kDa), and then it easily filtrated by the glomerulus being almost totally uptaken in the S1 segment of proximal tubule, in a process mediated by megalin and cubilin [25]. Approximately 10% is reabsorbed in distal tubule [26].

**Figure 4:**
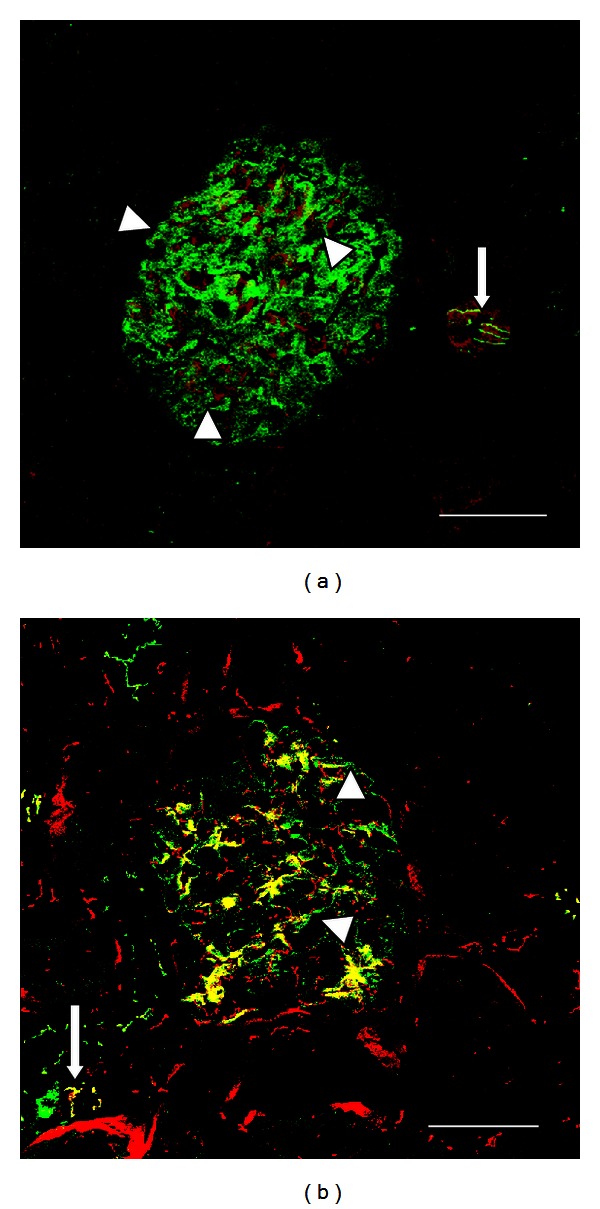
Comparative claudin 5 distribution in tubules and vessels in kidney from a control rat and a dam rat intoxicated with Cd during pregnancy. In renal sections from a normal dam rat claudin 5 (green label) costained with VE-cadherin (red label) is expressed in glomerular capillaries (arrow heads (a)) and in surrounding vessels (arrow, (a)). Expression of claudin 5 at the glomerular capillaries (arrow heads, (b)) and in surrounding vessels (arrow, (b)) is severely altered in a dam rat exposed to Cd. Bar = 50 *μ*m.

Cadmium induces cellular damage through several mechanisms, one of the best characterized is that it accumulates in mitochondria, where it blocks the respiratory chain at complex III resulting in an increased production of free radicals that enhances caspase activity leading to apoptosis [27]. Free Cd also binds to chemical functional groups in several proteins. It has been shown that Cd affects the distribution of paracellular TJ proteins decreasing the TER [28] (Figure 4).

The best characterized clinical findings of Cd renal toxicity are low molecular weight proteinuria, aminoaciduria, bicarbonaturia, glycosuria, and phosphaturia [29]. Also, it has been observed pulmonary obstructive disorders and different types of neoplasias, such as bladder and lung carcinomas [29–31].

Nephrotoxic effects are severe and include the development of a Fanconi-like syndrome, with glucosuria, aminoaciduria, and phosphaturia. Hazen-Martin et al. [32] reported in cultures of human proximal cells exposed to Cd, alterations in TER and in the structure of apical cell membrane junctions, latest observed by freeze-fracture microscopy. Adult rats exposed to Cd showed alterations in claudin 5 pattern in glomeruli, indicating that in addition to proximal tubule, this metal also damages glomerular endothelial cells. As expected, claudin 2 showed severe disruption of the “chicken fence” pattern in proximal tubules of animals intoxicated with Cd. Zinc administration partially protected against deleterious effects of Cd [33]. Prozialeck et al. [34] reported damage induced by this metal, to N-cadherin, E-cadherin, and *β*-catenin. It is interesting that Zimmerhackl et al. [35] reported that Cd is more toxic to LLC-PK1 cells (from porcine origin) than in MDCK cells (from canine origin), acting on the cadherin-catenin complex. These findings suggest that Cd toxicity may elicit differences related to species. Chronic exposure to Cd leads to hypertension and renal failure. Jacquillet et al. [28] described severe alteration in the pattern of claudin 5 in glomeruli from adult offsprings exposed *in uterus* to Cd. After birth those animals did not receive additional doses of this metal and their blood pressure and renal function were normal. However, they developed hypertension and renal damage in adult life. These animals also depicted alteration of the pattern and distribution of claudin 2 in proximal tubules. These findings showed the risk of in utero exposition to Cd on the development of arterial hypertension and renal damage in adult life. Taking into consideration that tobacco smoke is one of the main sources of contamination by Cd and the long lasting half-life of this metal in humans (10 to 30 years), exposure during gestation might be a critical hazard for hypertension and renal damage in offsprings [28].

### 3.3. Chromium

This metal is accumulated in the kidney, as other heavy metals. The toxic effects of this accumulation have been recognized mostly in acute intoxication episodes. Much less is known for chronic exposure. In a model of acute renal failure induced by potassium dichromate, Perez et al. [36] reported that abundant death renal cells were found in urinary sediment. Even more relevant was the finding of alive cells in urine. This finding suggested that the mechanisms, responsible for maintenance of cells attached to basal membrane and to neighbor cells (tight junctions, desmosomes, and gap junctions), were altered by this metal. Chromium (Cr) induces oxidative stress and it has been reported that oxidative stress damages TJs function. In the same study it was observed that administration of all-trans-retinoic acid, the active form of vitamin A, reduced the elimination of living cells [36]. Arreola-Mendoza et al. [37] reported that in addition to damage to proximal tubules demonstrated by increased excretion of glucose and decreased capacity of the secretory pathway of organic anions, distal functions of the nephron, as water regulation, were altered by this metal. Arreola-Mendoza et al. [38] confirmed in the same model that Cr severely altered expression and distribution of occludin and claudin 2; *α*-tocopherol, active form of vitamin E, protected renal cells from damage induced by this metal, through the participation of extracellular signal-regulated kinase 1/2 (ERK1/2).

Exposure to hexavalent chromium (Cr^6+^) causes mutagenic, carcinogenic, and toxic effects, some of which have been associated with its oxidative capacity. In the kidney, TJs are especially sensitive to oxidative stress. In this sense, Basuroy et al. [39] demonstrated that changes in the tubular oxide-reductive environment change the TJ proteins distribution affecting its permeability properties. Subsequently, Arreola-Mendoza et al. [38] and Perez et al. [36] showed, in rats exposed to Cr^6+^, proximal and distal tubule dysfunction, decreased glomerular filtration, as well as increased oxidative damage.

**Figure 5:**
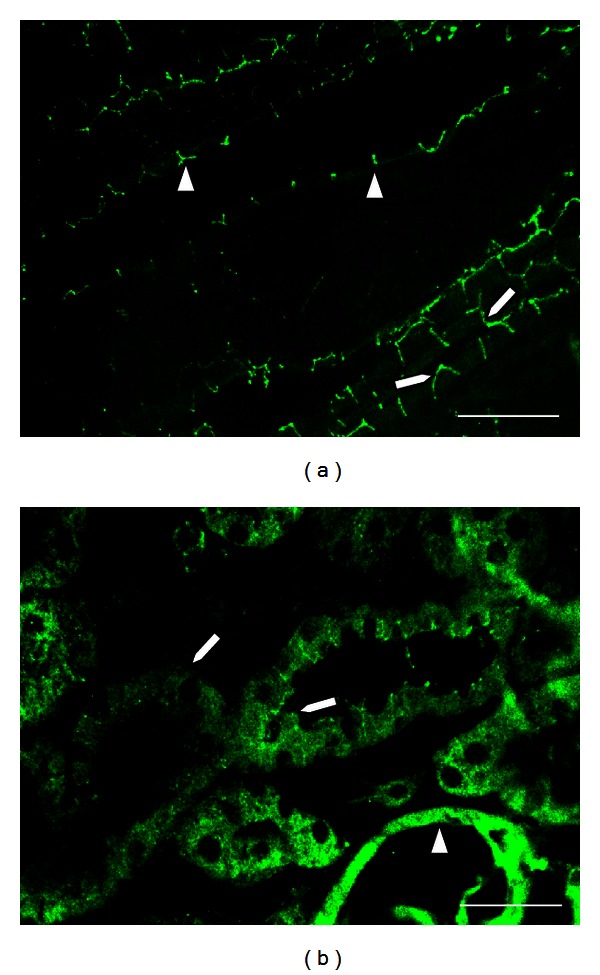
Comparative occludin distribution in tubules and vessels in kidney from a control rat and a rat treated with potassium dichromate. In control rats, occludin in vessel shows a clearly defined endothelial punctuated label (arrow heads, (a)). In tubules, label for occludin shows a typical “chicken fence” distribution (arrows, (a)). Rats treated with potassium dichromate (15 mg/kg, single dose) show severe alteration of occludin distribution in tubules (arrows, (b)), and in a vessel (arrow head, (b)). Bar = 50 *μ*m.

Chromium induces variations in serum creatinine, creatinine clearance (C_cr_), and fractional excretion of sodium (FeNa), and the latest can be mediated by the mislocation of claudin 2 and occludin induced by Cr [36] (Figure 5). These data implicate that oxidative stress induced by heavy metals affects the distribution of TJ proteins in proximal and distal tubules altering its permeability properties.

### 3.4. Lead

Lead nephrotoxicity has been recognized for more than a century; however, cell mechanisms involved have not been fully disclosed. This is more evident in the case of effect on intercellular junctions. Navarro-Moreno et al. [40] showed alterations in the intercellular apical region of proximal tubules from rats intoxicated during seven months with Pb. Glycosuria, aminoaciduria, hematuria, oxidative stress, and loss of apical microvilli were also observed. Lead shares with Cd the toxic effect of inducing arterial hypertension and vascular alterations, including glomerulonephritis [41, 42].

Lead is one of the most frequent divalent metals that induces nephrotoxicity. It has been demonstrated that even at low doses, Pb increases cardiovascular morbility [43].

Although, the maximum nontoxic dose of Pb blood levels has not been established, there is increasing evidence that even previously considered nontoxic Pb levels increase morbility and mortality rates in general population [43].

Once absorbed by intestine, lung and to a lesser extent through the skin, Pb binds to erythrocyte proteins at above 90%, and then it is distributed to soft tissues and bone. The latest is the main reservoir for Pb in the body. Its bloodstream levels increase during augmented bone turnover, notably during adolescence and pregnancy [44].

Lead binds to low molecular weight proteins in a proportion lower than 1%. For this reason it is freely filtered at the glomerulus and it is reabsorbed in the proximal tubular cells by a mechanism mediated by endocytosis. Once into the cell, Pb induces mitochondrial damage, uncoupling of respiratory chain, intracellular depletion of GSH, oxidative stress, and apoptosis [45].

It has been demonstrated that Pb enhances proinflammatory processes through activation of nuclear factor kappa B (NF*κ*B), which in turn activates intrarenal renin-angiotensin system and thus activates macrophages, generating an interstitial inflammatory process into the kidney [46]. In endothelial cells, the increased production of free radicals elicited by Pb inactivates vasorelaxant mediators such as nitric oxide (NO). These effects may partially explain the hypertension found in Pb intoxication [47].

The clinical findings of Pb toxicity can be divided in two categories depending on duration of exposure; acute toxicity manifested as aminoaciduria, glycosuria, hyperphosphatemia, haemolytic anemia, gout, and encephalopathy [48]; and chronic toxicity depicted as tubule-interstitial nephritis and progressive decrease of renal function. The toxic effects of Pb on proximal tubular epithelial cells decrease the urate excretion resulting in increased urate levels in the bloodstream [40, 49].

### 3.5. Mercury

Effects of Hg on proximal tubule were recognized long time ago leading to its use as diuretic. Recognition of its toxic effects precluded this therapeutic application. To our knowledge to date there is no available information on the effects of Hg exposure on intercellular renal TJs. As in the case of Cr toxicity, in Hg-intoxicated rats vitamin E protected against the renal damage and development of interstitial renal fibrosis [50].

Mercuric chloride (HgCl_2_) causes acute oxidant renal failure that affects mainly proximal tubules. Proteinuria induced by chronic exposure to Hg and the relationship between urinary Hg and renal damage were explored in rats. The results showed that the primary site of damage was the proximal renal tubule and that the glomerulus was eventually involved, due to the capability of Hg to be freely filtered at the glomerulus. The tubular ultrastructural analysis revealed that the lysosome was the most sensitive organelle to Hg, and there was a close relationship between the excretion of urinary Hg and the Hg detoxication mechanisms of the kidney. There is evidence that Hg was accumulated also in endothelia and mesangia of the glomeruli [51].

The mechanisms involved in renal damage (membranous nephropathy) induced by Hg are in relationship with the deposit of immune complexes directed both against laminin *β*1 and several complement factors. It has been previously reported that this mechanism can be mediated by the release of interferon-gamma (IFN-*γ*) and interleukin-4 (IL-4) from infiltrating glomerular macrophages which exerted toxic effects, resulting in a rapid decrement of TER of confluent monolayers. In addition, it has been demonstrated that podocytes exposed to IFN-*γ* and IL-4 also altered vimentin and laminin expression; indeed both of them interfered with monolayer integrity when added to the basolateral side of podocytes, indicating a selective damage [52]. Indeed, experimental models of HgCl_2_-induced renal glomerular injury showed increased deposits of fibronectin and lipids and enhanced cellularity in glomeruli [53].

Experimental models of HgCl_2_-induced renal glomerular injury showed alterations in urine osmolality, volume, and protein levels were seen within 24 h in response to 1 mg/kg of HgCl_2_ [54].

### 3.6. Bismuth

Toxicity of this metal on renal attachment mechanisms has been only scarcely reported. Acute renal failure induced by bismuth (Bi) has been reported [55]. This issue is relevant taking into consideration the wide use of Bi compounds to peptic ulcers treatment and to eradicate *Helicobacter pylori*. Loss of renal epithelial cell adhesion by selective N-cadherin displacement was reported by Leussink et al. [56]. They found that this effect was present both, *in vivo* and *in vitro* models. After 1 h treatment, N-cadherin had disappeared from adherent junctions of vital proximal tubular cells, whereas ZO-1, a TJ protein, remained present. Similar results were obtained in two cell lines derived from proximal tubules (NRK-52E and LLC-PK1) [57]. Interestingly, Bi salts have been found to be protective against cisplatin nephrotoxicity [57].

### 3.7. Nickel

Nickel compounds are associated with several human diseases mainly in lung and kidney cancers [58]. Vyskocil et al. [59] showed that even at low oral doses, soluble Ni induced changes in glomerular permeability in female laboratory animals, or enhanced the normal age-related glomerular nephritic lesions. Compared with male, female rats seem to be more sensitive to the nephrotoxic effect of Ni [59].

Subsequently, Horak and Sunderman [60] demonstrated that rats exposed to inhalation of Ni carbonyl display an increased urinary protein excretion. Among the acidic amino acids and amides, only the excretion of glutamic acid was increased. Urinary excretion of ammonia was greatly increased after exposure to Ni carbonyl. The alterations of urinary excretions of amino acids were apparently mediated by nephrotoxicity rather than by mobilization of amino acids from tissues, since plasma concentrations of amino acids were not significantly affected by exposure to Ni carbonyl. The pronounced diminution of glutamine excretion and the marked increase of ammonia excretion were consistent with enhanced renal production of ammonia from glutamine by action of glutaminase [60].

Accordingly, administration of Ni (6 mg per kg, i.p., three days) significantly enhanced the urinary excretion of alkaline phosphatase (ALP), lactate dehydrogenase (LDH), glutamate oxaloacetate transaminase (GOT), amino acids, and proteins. In addition, it increased the activity of serum ALP, GOT, and glutamate pyruvate transaminase (GPT) [61].

## 4. Mechanisms of Heavy Metals-Induced Nephrotoxicity

Renal toxicity induced by heavy metals has been recognized for over a century; however, the intracellular mechanisms of this nephrotoxicity remain unclear. Recent studies performed *in vivo* and *in vitro* (renal cell lines and isolated mitochondria) have indicated that oxidative stress, apoptosis, and necrosis are common phenomena in the course of nephrotoxicity elicited by these metals [62]. In recent years, the pathogenetic mechanisms of acute kidney injury (AKI) have been associated with endothelial and epithelial cell injury. Endothelial damage in glomeruli leads to proteinuria. Tubular cells undergo alteration of cell polarity, mislocalization of TJ proteins, and membrane transporters and finally develop necrosis and apoptosis. A common event in the action of all heavy metals in the proximal tubular cells is generation of oxidative stress that is manifested by (a) depletion of intracellular GSH and free radical scavenger levels, (b) inhibition of the activity of several antioxidant enzymes that participate in the detoxication of free radicals, and (c) increased reactive oxygen species (ROS) production.

Other phenomena such as the loss of the function of transporters, ATPases and ion channels, deranged metabolism, cytoskeleton, and cell polarity destabilization with loss of cell membrane integrity are present. Increased synthesis of MT proteins, upregulation of heat shock proteins (Hsp), increase in cytoplasmic concentration of Ca^2+^, impaired endocytosis, enhancement of ion conductances, and structural and functional damage in mitochondria have been described in the nephrotoxicity induced by heavy metals such as Cd, Hg, and Cr.

In this review we focus on the deleterious effects induced by heavy metals on the renal TJ structure and function and the participation of oxidative stress and renal cells response to these toxicants. It is known that TJ structure is compromised under oxidative stress conditions [71]. Nevertheless, there are few reports on the effects of heavy metals on the expression of renal claudins, occludin and ZO proteins, and the functional consequences. *In vitro* studies have shown that oxidative stress induced by H_2_O_2_ disrupts the TJs of cultured MDCK cells through the activation of G protein *α*12 [72].

Multiple pathways participate in Cd-induced nephrotoxicity [73]. Cd exposure causes Ca^2+^ release from the endoplasmic reticulum, and activation of ERK and depolarization of the mitochondrial membrane potential, which induces autophagy and apoptosis, leading to the death of mesangial cells. Thus, the calcium-mitochondria-caspase signaling pathway contributes significantly to the Cd-induced death of mesangial cells [73]. Another mechanism involved in Cd toxicity is the production of ROS, which in turn activates glycogen synthase kinase (GSK-3*β*) leading to autophagy and cell death. ROS production plays a pivotal role in the pathogenesis of several kidney diseases, and Cd might act on the mitochondria to induce the release of ROS.

Cadmium can selectively damage the TJ in LLCPK1 cultures [34, 35], and in toad epithelial (A6) cells [74], with decrements of TER without affecting cell viability. Jacquillet et al. [33] reported that Cd intoxication alters the renal expression and localization of claudin proteins in rats. The same group reported that exposure of rats to Cd during gestation disrupts claudins 2 and 5 in the kidney of the adult offspring [28]. In conclusion, in utero exposure of Cd leads to toxic renal effects in adult offspring. Cd-induced contraction of both mesangial cells and isolated rat glomeruli occurs through activation of p38 mitogen-activated protein kinase (p38 MAPK) [75]. It has been described that exposure of MDCK cells to CdCl_2_ caused a robust increase in cellular levels of Hsp70 and eliminated vectorial active transport by proximal tubular cell lines. However, Cd exposure did not induce alterations in occludin expression [76].

Cr^6+^ compounds are oxidizing agents that directly induce tissue damage. The kidney is the main target for Cr accumulation, which might result in acute tubular necrosis in humans after oral or dermal absorption [77]. ROS production, DNA damage, and apoptotic cell death play a pivotal role in the nephrotoxicity induced by Cr^6+^ [63]. In a model of acute kidney injury induced by Cr^6+^, it was shown that oxidative stress disrupts distribution of occludin and claudin 2, and that the treatment with the antioxidant *α*-tocopherol prevents these changes, through ERK 1/2 pathway [38]. There are reports on the toxicity of Cr on the TJ structure in other organs; Murthy et al. [78] reported that daily administration of Cr^6+^ in adult rats for 15 days produced significant increases in the blood and testicular Cr levels, and lanthanum perfusion in treated rats revealed leakage at Sertoli-cell TJs.

The effects of Hg are highly dependent on the different chemical forms of this metal. Dental amalgam is a major source of Hg ingestion [79]. In humans and other mammals, kidneys are the primary targets for accumulation of mercuric ions after exposure to elemental or inorganic forms of Hg [80]. *In vitro* studies have shown that Hg exposure decreased TER in confluent monolayers of podocytes in which IFN-*γ* and IL-4 expression are increased, which was closely associated with altered immune-reactivity of the TJ protein ZO-1 [52]. Kawedia et al. [81] reported that Hg-treated salivary epithelial cells showed a decreased TER, Hg^2+^ triggered phosphorylation of occludin via a protein kinase A (PKA) dependent mechanism decreasing TJ expression of occludin and increasing the paracellular permeability. The actions of two Hg compounds, the inorganic HgCl_2_, and the organic methyl mercury chloride (MeHg), on ion transport across the rat colon, were studied by Bohme et al. [82]; they found that Hg compounds, administered to the luminal side, induced a large, concentration-dependent increase of tissue conductance. The transepithelial movement of the extracellular marker, mannitol, was enhanced in the presence of the Hg compounds, indicating that they cause an increase in the paracellular permeability of the epithelium.

Renal toxicity by Pb was recognized in 19th century; however, the cellular mechanisms involved in the renal damage induced by chronic exposure to this metal remain largely unknown. Navarro-Moreno et al. [40] described alteration of intercellular junction at the proximal tubules of rats intoxicated with Pb for seven months. In addition, they described oxidative damage, glucosuria, proteinuria, and morphological changes in mitochondria, nuclei, lysosomes, and loss of apical microvilli. Environmental Pb intoxication causes irreversible neurological disturbances by mechanisms remaining to be identified. It has been reported that Pb induced a reduction in the expression of occludin in the TJ of the blood-brain barrier of young rats altering the paracellular permeability of this structure [83]. Another study performed by Shi and Zheng [84] showed that early exposure to Pb (prior to the formation of tight barrier) significantly reduced the tightness of blood-cerebrospinal fluid barrier (BCB), as evidenced by reduction in TER and increase in the paracellular permeability of ^[14]^C-sucrose. Exposure to Pb after the formation of tight barrier, however, did not cause any detectable barrier dysfunction. In the same study, Pb exposure decreased both the mRNA and protein levels of claudin 1. Pb exposure, however, had no significant effect on ZO-1 and occludin expression. These data suggest that Pb exposure selectively alters the cellular level of claudin 1, which, in turn, reduces the tightness and augments the permeability of tight BCB.

## 5. Oxidative and Nitrosative Stress in Kidney

Oxidative stress is a state in which the cell undergoes altered intracellular redox homeostasis; that is, the balance between oxidants and antioxidants is lost. This imbalance is caused by excessive production of ROS and/or deficiency in antioxidant mechanisms. ROS and reactive nitrogen species (RNS) often act together to create a state of oxidative stress. The three molecular targets more susceptible to be damaged by ROS are DNA, lipids, and proteins [85]. Protection against oxygen free radicals involves enzyme activity: catalase (CAT), superoxide dismutases (SOD), glutathione peroxidase (GPx), glutathione reductase (GR), and nonenzymatic (vitamin E, vitamin C, GSH) systems of protection [86–89]. Balanced combination of the actions of antioxidant enzymes such as SOD, GPx, and CAT is very important to achieve a better protection against oxidative stress damage [90].

Oxidative stress has been identified as one of the factors that trigger certain pathologies such as asthma, cardiopulmonary disorders, hypertension, Parkinson, and Alzheimer and plays an important role in the development of kidney diseases [91–94]. A significant imbalance in the activities in pro-oxidants and antioxidants in patients with renal dysfunction has been reported [95]. In patients with acute renal failure, generation of ROS is increased due to a deficiency of the major antioxidant systems. In these patients increased oxidation of proteins occurs in plasma, mainly albumin (carbonylation and disulfide bond formation). Other complications, such as tissue damage, favour severe irreversible kidney damage [96–98].

### 5.1. Metal-Induced Toxicity and Carcinogenicity by the Generation of ROS and RNS

Metal-mediated formation of free radicals causes various modifications to DNA bases, augmented lipid peroxidation, and altered Ca^2+^ and sulfhydryl homeostasis. Lipid peroxides, formed by the attack of radicals on polyunsaturated fatty acid residues of phospholipids, can further react with redox metals, finally producing mutagenic and carcinogenic malondialdehyde, 4-hydroxynonenal, and other exocyclic DNA adducts (etheno and/or propano adducts). Whilst Fe, Cu, Cr, Vanadium (V), and Co undergo redox-cycling reactions, for a second group of metals, Hg, Cd and Ni, the primary route for their toxicity is depletion of glutathione and binding to sulfhydryl groups of proteins. Arsenic (As) is thought to bind directly to critical thiols; however, other mechanisms, involving formation of hydrogen peroxide under physiological conditions, have been proposed. The unifying factor in determining toxicity and carcinogenicity for all these metals is the generation of ROS and RNS. Common mechanisms involving the Fenton reaction, generation of the superoxide radical (O_2_^•−^), and the hydroxyl radical (OH^•^) appear to be involved for Fe, Cu, Cr, Vanadium, and Co, primarily associated with mitochondria, microsomes, and peroxisomes damage. Nitric oxide seems to be involved in arsenite-induced DNA damage and pyrimidine excision inhibition.

Lipids in the kidney suffer damage due to oxidative stress generated by exposure to certain metals such as Cr (Table 1). Lipoperoxidation is produced in different regions of kidney and it is attenuated by the *α*-tocopherol [37, 38].

Renal tissue has high requirements for metabolic energy and relies heavily on aerobic metabolism for the production of ATP through oxidative phosphorylation. The reduction of molecular O_2_ along the electron transport chain within mitochondria is vital for renal cellular function, yet with a potentially devastating long-term effect [99]. Oxidative and nitrosative stress, by reducing the activity of complexes III, IV, and V of the respiratory chain and decreasing ATP levels, might contribute to mitochondrial dysfunction [100]. Mitochondrial injury leads to mitochondria dysfunction and perpetuated oxidative stress. The loss of mitochondrial membrane potential causes release of cytochrome-C and activation of caspase pathways that causes apoptotic detachment of renal cells. Removal of dysfunctional mitochondria through autophagy leads to “autophagic cell death” or apoptosis [101].

Chromium also causes damage to the kidney through oxidative stress. Potassium dichromate administered intraperitoneally to Sprague-Dawley rats for 5 days at doses of 2.5, 5.0, 7.5, and 10 mg/kg body weight per day caused a significant increase of ROS production in both liver and kidney, with increments in superoxide dismutase and catalase activities and DNA damage [63].

Potassium dichromate (K_2_Cr_2_O_7_) is a chemical compound that is widely used in metallurgy, chrome plating, chemical industry, textile manufacture, wood preservation, photography, photoengraving, stainless steel industry, and cooling systems. Its nephrotoxicity is associated with oxidative and nitrosative stress [102].

Cd is a toxic trace metal that is absorbed through lung, gastrointestinal tract, and skin. Following absorption, Cd is taken up by the hepatocytes and also circulates in blood, bound to MT. The Cd-MT complex, due to its small molecular size, is easily filtered through the glomerular membrane and taken up by renal tubular cells. In the cell this complex induces oxidative stress by interaction of Cd with mitochondrial structures and causes renal damage [103]. The intoxication of Cd leads to enhanced lipid peroxidation and protein carbonylation and to decrease of SOD activity and GSH levels [70] (Table 1).

Hg and its compounds are environmental and industrial agents that induce severe nephrotoxicity, both in man and animals. HgCl_2_ administration is a classic model for the study of the pathogenesis of inorganic Hg toxicity, both *in vitro* and *in vivo* systems, as reviewed by Zalups [80]. HgCl_2_ affects cell oxidative function due to its high affinity for cellular cysteine thiols [104]. Hg nephrotoxicity is characterized by altered antioxidant enzymes [105], lipid peroxidation [106], reduced ATP content [107] and it leads to tubular epithelium necrosis, even after a single exposure [108]. HgCl_2_-induced damage is strictly dependent on the route of administration, time, and dose [109]. The nephrotoxic dose of inorganic Hg is significantly different in various species. In humans it is fatal in the range of 10–42 mg/kg [110], while in mice the oral lethal dose 50 (LD50) is higher, between 25.9 and 77.7 mg/kg [111]. McDowell et al. [112] reported progressive morphological and functional alterations from 6 h on in the straight portion of rat proximal tubules after a dose of 4 mg/kg HgCl_2_. After administration to rats of 1.5 mg/kg HgCl_2_, Girardi and Elias [113] reported an increase in glutathione reductase and peroxidase and reduced urinary volume. Severe tubular necrosis was detected after 16 h in rat proximal tubules and after 48 h in the entire cortical area.

Exposure to Pb is ubiquitous, with the highest levels found in some environmental exposures that have been associated with a number of serious systemic adverse effects involving the nervous system, blood-forming organs, lungs, and kidneys. Pb is also one of the most prevalent and nephrotoxic metals to human. It induces oxidative stress conditions in specific organ targets not only through generation of ROS but also by decrement of cellular antioxidant mechanisms [114] (Table 1).

**Figure 6:**
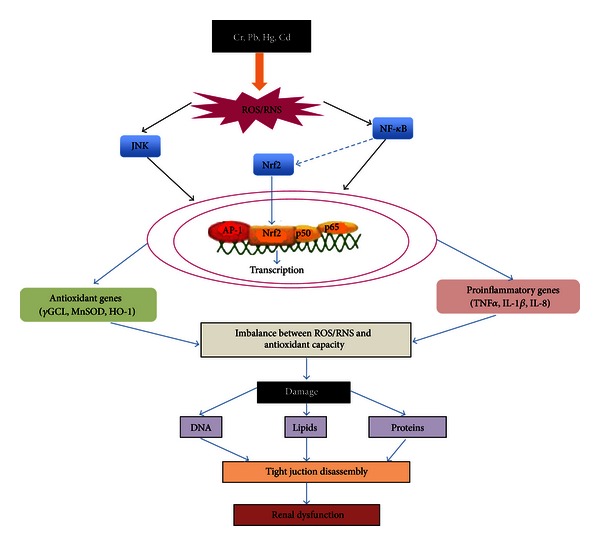
Mechanisms of oxidant stress and nephrotoxicity induced by heavy metals. Metal exposure produces an increase in reactive oxygen species (ROS) and reactive nitrogen species (RNS) activating signaling pathways, such as nuclear factor kappa B (NF*κ*B), nuclear factor (erythroid-derived 2)-like 2 (Nrf2), and c-Jun N-terminal kinase (JNK) generating the activation of antioxidant response, as well as inflammation. This response elicits oxidative and nitrosative stress, which damages DNA, lipids, and proteins. One of the consequences of oxidative stress is the disruption of the tight junction (TJ) proteins that may cause renal dysfunction.

A proposed mechanism through which heavy metals disrupt the structure and function of the TJ and cause renal dysfunction is shown in Figure 6.

## 6. Conclusion

Renal toxicity induced by heavy metals is a circumstance that may occur at any age and disrespect of gender. Acute exposition may cause renal failure, but chronic exposure results in a great variety of alterations, depending on the dose and duration of exposure. Occupational and environmental sources are of relevance. Damage induced by heavy metals on the proteins that constitute TJs is under current investigation, as well as the severe physiological consequences. It should be emphasized that most heavy metals depict oxidative stress in the kidney and this is one of the mechanisms involved in disruption of TJs. Therefore, to disclose the role of antioxidants in the prevention of oxidative stress might prove helpful in the prevention of the disassembly of the TJ structure.

## Figures and Tables

**Table 1:** Biomarkers of oxidative stress and antioxidant capacity in heavy metals-induced nephrotoxicity.

Metal	Damaged target	Biomarkers of oxidative stress	Biomarkers of antioxidant capacity	Sources
Cr^6+^ (*In vivo*)	Lipid peroxidation DNA Protein carbonylation	MDA TBARS Urinary NO^2−^/NO^3−^ 3-NT	SOD CAT GPx GR HO-1	[37, 38, 63–66]

Pb (*In vivo*)	Lipid peroxidation	TBARS	GSH GSSG CAT	[67]

Pb and Hg (*In vitro*)		MTs	GSH	
	Mitochondria	Hsp25	GST	[68]
	Apoptosis	Hsp72		
		Grp78		

Hg (*In vivo*)		MTs	MEL	
	Mitochondria	Hsp72		[69]
		Grp75		
		iNOS		

Cd *(In vivo) *	Lipid peroxidation Protein carbonylation	TBARS DNPH assay	SOD GSH CAT	[70]

CAT: catalase; DNPH: 2,4-dinitrophenylhydrazine; GPx: glutathione peroxidase; GR: glutathione reductase; Grp: glucose-regulated proteins; GSH: reduced glutathione; GSSG: oxidized glutathione; GST: glutathione-S-transferase; HO-1: Heme oxygenase-1; Hsp: heat shock proteins; iNOS: inducible nitric oxide synthase; MDA: malondialdehyde; MEL: melatonin; MT: metallothionein; NO^2−^/NO^3−^: nitrate/nitrite; 3-NT: 3-nitrotyrosine; SOD: superoxide dismutase; TBARS: thiobarbituric acid reactive substances.
